# Molecules in pain and sex: a developing story

**DOI:** 10.1186/s13041-017-0289-8

**Published:** 2017-03-07

**Authors:** Josiane C. S. Mapplebeck, Simon Beggs, Michael W. Salter

**Affiliations:** 1 Program in Neurosciences & Mental Health, Hospital for Sick Children, Toronto, ON Canada; 2 Department of Physiology, University of Toronto, Toronto, ON Canada; 3 University of Toronto Centre for the Study of Pain, Toronto, ON Canada; 4 Developmental Neurosciences Programme, UCL Great Ormond Street Institute of Child Health, London, UK

**Keywords:** Sex differences, Microglia, Neuropathic Pain, T cells, Spinal Cord

## Abstract

Microglia are dynamic immune cells with diverse roles in maintaining homeostasis of the central nervous system. Dysregulation of microglia has been critically implicated in the genesis of neuropathic pain. Peripheral nerve injury, a common cause of neuropathic pain, engages microglia-neuronal signalling which causes disinhibition and facilitated excitation of spinal nociceptive pathways. However, recent literature indicates that the role of microglia in neuropathic pain is sexually dimorphic, and that female pain processing appears to be independent of microglia, depending rather on T cells. Despite this sex difference, pain signalling in the spinal cord converges downstream of microglia, as NMDAR-mediated facilitated excitation in pain transmitting neurons is consistent between males and females. Determining whether pain signalling is sexually dimorphic in humans and, further, addressing the sex bias in pain research will increase the translational relevance of preclinical findings and advance our understanding of chronic pain in women.

## Introduction

Chronic pain is a highly prevalent and economically costly health condition [1, 2]. In the United States, at least 116 million adults have chronic pain with an estimated annual cost of $560-$635 billion [1]. Neuropathic pain, a common form of chronic pain, is caused by a lesion to or disease in the peripheral or central somatosensory nervous system [3]. Neuropathic pain is characterized by spontaneous pain, hyperalgesia and allodynia (pain hypersensitivity) [4]. Spontaneous pain is persistent or paroxysmal pain that is not elicited by a stimulus. Hyperalgesia is increased pain response to a noxious stimulus while allodynia (pain hypersensitivity) is pain in response to a normally innocuous stimulus.

Preclinical studies using rodent models of neuropathic pain have implicated spinal microglia as key mediators of peripheral nerve injury (PNI)-induced pain hypersensitivity [5–7]. However, evidence suggests that the role of microglia in neuropathic pain is sex-dependent [8]. This sexual dimorphism was only recently discovered as preclinical pain research has generally excluded female subjects. The consensus was that oestrus caused increased variability, now shown not to be the case [9, 10]. Consequently, the overwhelming majority of preclinical pain research is conducted using only male rodents [11], a sex bias reflective of the biomedical field as a whole [12]. This sex bias poses serious issues for the translational relevance of preclinical pain research given that women represent the majority of patients with chronic pain, including neuropathic pain [2, 13, 14]. Additionally, women appear to be more sensitive to experimentally induced pain [15, 16]. Sex differences in response to pain treatment in humans have also been reported [16], such as increased morphine analgesia in females [17].

## Microglia ontogeny and function

Microglia constitute an estimated 10% of centrally located cells and are the principal immune cell of the central nervous system (CNS) [18, 19]. As such, microglia maintain physiological homeostasis by responding directly to insults to CNS integrity such as traumatic brain injury, toxins, pathogens or other physiological stressors. Threats to the CNS produce characteristic microglial responses including proliferation, changes in morphology, antigen presentation and release of pro-inflammatory signalling molecules such as cytokines [19, 20]. Microglia share functional similarities to macrophages, which are innate immune cells involved in peripheral inflammation, but are distinct in cellular origin [21]. Microglia derive from primitive myeloid progenitors which originate in the yoke sac prior to embryonic day 8 and populate the developing CNS [22, 23]. Microglia cell populations are maintained and proliferate via self-renewal, without recruitment of peripheral precursors from the blood [24, 25].

Microglia were considered quiescent in the absence of CNS damage, reflected in classification of cells with long, ramified processes and small cell bodies as ‘resting’ microglia. In fact, microglia are highly dynamic with motile processes which extend and retract rapidly over a period of seconds to minutes within non-overlapping microdomains [7, 26, 27], providing constant environmental surveillance in addition to injury response. Consequently, CNS insult induces an immediate tropic response towards the injury site mediated by extracellular ATP signaling via P2Y12 [26, 28]. The physiological functions of microglia have now been shown to be far more diverse, with fundamental roles in ensuring healthy CNS functioning through phagocytic clearing of cellular debris, responding to and modulating neuronal activity, influencing synaptic pruning and maturation as well as modulating synaptic plasticity [29–34]. Misregulation of this normal dynamic functioning of microglia can contribute to pathology associated with disease or injury, and a prime example of this is the role of microglia in nerve injury-induced chronic pain.

## Spinal microglial reactivity after injury to peripheral nerves

**Fig. 1:**
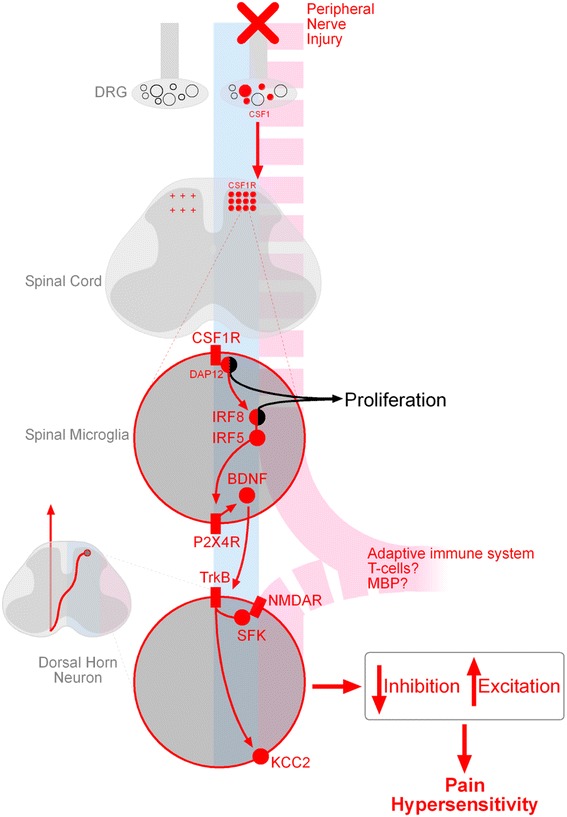
Schematic showing the cellular and molecular pathway involved in neuropathic pain following peripheral nerve injury (*top*). The presence and/or relevance of the pathway in males and females is shown where elements of the pathway lie on the *blue* (male) and *pink* (female) lines. A *solid line* indicates evidence exists for involvement in that sex; a *broken line* indicates either absence of evidence or yet to be tested. Pathway components in *red* show known involvement in spinal changes leading to reduced inhibition, increased excitation and resultant increase in pain hypersensitivity. See text for further details

PNI produces a stereotypical response in spinal microglia characterized by proliferation around central terminals and cell bodies of the peripherally damaged sensory and motor nerves respectively. The proliferative response is dependent on resident spinal microglia, as there is little, if any, infiltration of bone marrow-derived cells [35] or monocytes [36] after peripheral nerve injury. Although a striking and consistent response after PNI, it is unknown whether microglial proliferation in the dorsal horn is necessary for PNI-pain hypersensitivity. But, it is clear that the proliferation of microglia per se is not sufficient for that development of PNI-induced pain hypersensitivity [36–39]. Thus, a proliferative microglial response should not be considered as a proxy measure of pain [40]. Proliferation is accompanied by retraction of ramified processes and adoption of an amoeboid morphology and changes in expression of cell-surface proteins [41]. PNI activates *de novo* colony-stimulating factor 1 (CSF1) production in injured sensory neurons, which is transported to the spinal cord and binds to CSF1 receptors on microglia [25]. CSF1 activity simultaneously engages a membrane adaptor protein DAP12-independent pathway responsible for microglial proliferation and a DAP12-dependent pathway mediating upregulation of microglial genes associated with pain hypersensitivity, including *Irf8* and *Irf5.* [25, 37, 42]. Increases in IRF8 expression after PNI activates IRF5 which binds specifically to the promotor of *P2rx4,* leading to an upregulation of P2X4 receptor (P2X4R) expression on microglia [37, 42] (see Fig. 1).

Similar to signals in ascending pathways such as CSF1, descending serotonergic signalling has also been implicated in inducing spinal microglia reactivity [43] and facilitation of pain hypersensitivity [43, 44]. Alleviation of pain hypersensitivity occurs after inhibition of spinal 5-HT_3_ receptors in nerve-injured animals [43]. Furthermore, intrathecal application of a 5-HT_3_ agonist results in glial reactivity and development of hypersensitivity in rats [43]. Additionally, spinal microgliosis due to peripheral inflammation can be attenuated by depletion of the 5-HT system [43].

## Microglia signalling in the spinal cord

Upregulation and activation of microglial P2X4R expression in the spinal dorsal horn has been found to be necessary for development of PNI-induced pain hypersensitivity [5, 7]: pharmacological suppression of spinal P2X4Rs attenuates PNI-induced pain hypersensitivity [5], and P2X4-deficient mice (*P2X*_*4*_^*−/−*^) show a lack of hypersensitivity after PNI [38]. In addition, adoptive transfer of ATP-stimulated microglia into naïve rats produces hypersensitivity [5] and sensitization of lamina I dorsal horn neurons [45] similar to that seen in nerve injured rodents. Activation of microglial P2X4Rs, by ATP released from dorsal horn neurons [46], evokes an influx of extracellular calcium, phosphorylating p38 MAP kinase and results in release of brain derived neurotrophic factor (BDNF) [6, 47]. Microglial BDNF secretion is impaired in P2X4-deficient mice [38]. Furthermore, deletion of microglial BDNF (CX_3_CR1^CreER^ x loxP-Bdnf) both prevents and reverses PNI-induced hypersensitivity in mice [8].

Secreted BDNF stimulates neuronal TrkB receptors in the dorsal horn initiating downregulation of potassium chloride co-transporter KCC2 and a consequent shift in the transmembrane anion gradient [6, 48]. KCC2 maintains neuronal chloride extrusion, ensuring low intracellular chloride concentrations [49]. γ-aminobutyric acid type A (GABA_A_) receptor functioning depends on the chloride gradient; thus, increased intracellular chloride due to KCC2 downregulation impairs GABA-mediated inhibitory synaptic transmission [50]. Pharmacological blockade or antisense knockdown of KCC2 produces hypersensitivity in naïve rodents [48]. Pharmacological activation of KCC2 reduces intracellular chloride concentration and alleviates hypersensitivity in nerve-injury models [51]. Reduced chloride extrusion capacity also promotes an efflux of HCO_3_^−^ anions through GABA_A_ channels which further enhances GABA-mediated disinhibition [52]. Carbonic anhydrase (CA) is a family of enzymes which catalyzes the synthesis of HCO_3_^−^ [53]. Inhibition of CA alleviates nerve-injury induced hypersensitivity, likely by decreasing the depolarizing efflux of HCO_3_^−^ [52]. The consequence of disinhibition is therefore a net increase in excitability of lamina 1 neurons, which transforms spinal output and produces the hallmark symptoms of neuropathic pain in rodents [45, 48].

In addition to disinhibition, facilitated excitation produced by Src kinase-mediated enhancement of NMDAR currents [54] contributes to hyperexcitability of lamina 1 projection neurons and associated pain hypersensitivity [55]. Src is a non-receptor protein tyrosine kinase with diverse physiological functions [56]. Src binding to the NMDAR complex via NADH dehydrogenase subunit 2 (ND2) increases NMDAR activity [57]. Enhancement of NMDAR function amplifies glutamatergic synaptic transmission, increasing the output of lamina 1 neurons. Uncoupling of Src kinase from the NMDAR complex blocks Src-mediated NMDAR activity enhancement [57]. Furthermore, intrathecal application of a peptide that disrupts Src binding alleviates nerve-injury induced pain hypersensitivity [55]. The factors mediating Src-ND2 binding after PNI are not fully understood. Microglial BDNF may be fundamental to this process as intrathecal BDNF in naïve rodents potentiates NMDARs in a Src family kinase-dependent manner [58] and potentiation of NMDAR currents due to nerve injury is dependent on BDNF signalling [59]. Cytokines can also enhance NMDAR function through Src activation [60]. Furthermore, chloride-mediated disinhibition is required for potentiation of NMDAR activity after nerve injury [59]. Thus, multiple signalling molecules may contribute to NMDAR-enhancement after PNI.

## Sexually dimorphic role of microglia in pain

The microglia-neuronal signalling pathway was established through experiments using almost exclusively male rodents; thus, its role in pain processing was not established in females. Recent experiments using mice of both sexes have shown that microglia are not involved in mediating pain hypersensitivity in females with PNI, suggesting the existence of sexually dimorphic pain processing [8].

The first indication of sexually dimorphic pain processing was evidence showing that spinal TLR4s, which are expressed specifically on microglia in the CNS, contribute to PNI-induced pain hypersensitivity in male but not female mice [61]. TLR4 is a member of the Toll-like receptor family involved in response to pathogens such as bacterial lipopolysaccharide and consequent activation of the innate immune system [62]. The sexually dimorphic role of TLR4 prompted broader investigation into the relevance of microglia to neuropathic pain in female mice. Damage to a peripheral nerve produces spinal microglial reactivity in female mice comparable to that of males [8, 63]. However, application of intrathecal minocycline, propentofylline or fluorocitrate, which may inhibit processes in glial cells, or specific microglial lesioning (via intrathecal injection of saporin toxin conjugated to the MAC-1 receptor) in mice alleviates nerve injury-induced pain hypersensitivity in males only and are entirely ineffective in females, suggesting that microglia are not necessary for pain hypersensitivity in female mice [8]. Inhibition of spinal P2X4Rs attenuates pain hypersensitivity in male but not female mice, confirming that microglia neuronal signalling does not contribute to pain processing in female mice [8]. p38 MAP kinase inhibition in the spinal cord is also ineffective in attenuating hypersensitivity in female mice [8]. While male microglial BDNF knockout mice (CX_3_CR1^CreER^ x loxP-Bdnf) display significant impairments in development and maintenance of PNI-induced hypersensitivity, pain processing is unaffected in female knockout mice [8]. Furthermore, pharmacological inhibition of spinal BDNF reverses hypersensitivity in males only, ruling out the possibility that non-microglia derived BDNF contribute to female pain processing [8]. Assessment of genes relevant to the microglia neuronal signalling pathway demonstrated that upregulation of *P2rx4* is exclusive to male mice, indicating that the P2X4R-dependent signalling pathway is not being engaged in female mice, which may be the key to the sex dependency of microglia in pain [8]. IRF8-IRF5 signalling lies upstream of P2X4R transcriptional upregulation [37, 42]; therefore, a sex difference in *P2rx4* levels after nerve injury may result from differential IRF8-IRF5 expression. However, PNI upregulates *Irf8* and *Irf5* expression equally in both sexes, which could account for the microglial proliferation observed in females [8]. Thus, the inference is that IRF5 mediated transcription of *P2rx4* is likely not occurring in females, which may be the key to the sex-dependency of microglia in pain.

Sexual dimorphism of the microglia-neuron signalling pathway has been confirmed elsewhere [64]. Inhibition of spinal p38 MAP kinase alleviates nerve injury-induced pain hypersensitivity in male but not female mice as well as rats, consistent with the more substantial p38 phosphorylation levels after injury in males [64]. Furthermore, spontaneous excitatory postsynaptic currents (EPSCs) are depressed only in male lamina IIo neurons during p38 MAP kinase blockade [64]. This sex difference appears to be spinally restricted as p38 MAP kinase inhibition through intraperitoneal and perineural application routes produces robust reversal in either sex [64]. The specificity of this sex difference is consistent with the sexually dimorphic role of spinal but not peripheral TLR4 in pain [61]. However, recent evidence has shown that spinal microglia are involved in mediating bone cancer pain in female rats [65], which suggests that sex differences in pain processing may not be consistent across injury models. The contribution of descending serotonergic circuitry to neuropathic pain in females has yet to be investigated, as previous work in investigating 5-HT_3_ was conducted using male rodents only [43, 44].

Despite the absence of a role for microglia in mediating neuropathic pain in females, there seems to be mechanistic convergence at the neuronal level as antagonising NMDAR activity alleviates pain hypersensitivity in both sexes [8]. This suggests that despite a sex difference in upstream signalling, similar neuronal changes occur after nerve injury, i.e. potentiation of synaptic NMDAR activity. Targeting convergent mechanisms between females and males is an alternative strategy to development of sex-specific therapies. It is still unknown whether the role for NMDARs in female pain processing is Src kinase dependent. Given the congruency in NMDAR involvement between females and males, it is possible that disinhibition due to KCC2 downregulation also contributes to pain behavior in both sexes. Investigation of whether impaired chloride extrusion mediates pain in females is critical given the interest in targeting KCC2 to treat neuropathic pain [51]. Orally administered drugs which rescue plasma membrane expression of KCC2 produce strong analgesia in rodent models of PNI without loss of motor function [51]. Such chloride extrusion enhancers remain to be tested in females. If KCC2-dependent disinhibition is consistent between the sexes, this would indicate that the sex difference in neuropathic pain processing is restricted to immune system functioning.

The underlying cause of the sexual dimorphism in pain processing remains unidentified; however, sex hormones represent a strong candidate [66]. Engagement of the microglial-dependent pathway appears to be contingent on the presence of high testosterone levels, regardless of sex [8]. Sex steroids have profound influences on immune functioning and may be responsible for many sex differences in pain, see Rosen et al., 2017 for a in-depth review of this topic [66]. Hormones also have been shown to regulate gene transcription [67, 68]. Thus, future investigation is required to determine whether sex steroids underlie the differential upregulation of *P2rx4* between males in females after nerve injury.

## Critical role for adaptive immune cells in females

That pain hypersensitivity in female mice is independent of microglia, which are innate immune cells, led to considering the possibility that cells of the adaptive immune system may be necessary in females [8]. It has been previously reported that male mice lacking adaptive immune cells develop less hypersensitivity after nerve injury [69, 70]. In contrast, it was found that nerve-injured adaptive immune cell knockout mice (B6.129S7-*Rag1* ^*tm1Mom*^/J and NU/J) display equivalent hypersensitivity to their wildtype counterparts, regardless of sex. However, interrogation of pain signalling mechanisms in these mice revealed that females lacking adaptive immune cells employ a microglia-dependent pathway in the mediation of pain hypersensitivity. Immune system reconstitution of female Rag1 knockouts with spleenocytes causes a ‘switch’ to a microglia-independent pathway. Therefore, it is hypothesized that the presence of adaptive immune cells, likely T cells, is necessary to drive the non-microglia pathway (Fig. 1). T cells migrate into the spinal cord after nerve injury and have been implicated as key regulators of hypersensitivity [69–71].

T cells of mouse and human lineage display a testosterone-dependent sex difference in expression of peroxisome proliferator activated receptors (PPARs), transcription factors integral in cytokine regulation [72]. Expression of PPARα is augmented by testosterone, which boosts secretion of interleukin-17A [72]. Conversely, testosterone decreases expression of PPARγ, which suppresses production of interferon-γ [72]. Infiltrating cells into the spinal cord after nerve injury appear to be primarily T helper type 1 (Th1) lymphocytes [73]. As interferon-γ is the prototypical Th1 cytokine, infiltrated T cells in nerve-injured male mice may secrete fewer pro-inflammatory mediators relative to females. Consistent with sexually dimorphic PPAR expression, intrathecal application of the PPARα agonist, fenofibrate, attenuates nerve injury-induced hypersensitivity in male mice only, a drug effect abolished by castration [8]. Furthermore, intrathecal administration of the PPARγ agonist, pioglitazone, reverses hypersensitivity after nerve injury in females, but not males [8]. Pioglitazone-mediated analgesia in females is attenuated by treatment with testosterone propionate [8]. In addition to sex differences in T cell phenotype, female mice also have higher peripheral and central T cell counts than male mice [8]. Combined, differential T cell numbers and cytokine secretion could lead to sexually dimorphic involvement of T cells in pain processing. A T cell driven mechanism could also directly supress the microglia-dependent pathway through inhibition of *P2rx4* transcription.

Hypersensitivity resulting from a sciatic nerve injection of myelin basic protein (MBP) in female rats is also mediated by T cells [74]. MBP has also been implicated in the development of PNI-induced hypersensitivity in females [75]. Thus, MBP-mediated activation of T cells may represent a component of the microglia-independent pathway. T cells could release pro-inflammatory cytokines to produce neuronal changes, such as enhancement in NMDAR functioning, which result in hyperexcitability of spinal nociceptive circuitry. B cells may also represent a critical mediator of the microglial-independent pathway. However, nerve injury does not induce spinal infiltration of B cells in male mice [70] and little evidence has implicated B cells in pain processing. The exact role for adaptive immune cells and MBP in mediating pain in females requires further investigation.

## Relevance to clinical pain in humans

The role of microglia in chronic pain in humans of either sex remains unclear. Significant differences exist in immune system functioning between rodents and humans, which poses issues for translation of preclinical findings to humans [76, 77]. Post-mortem analysis of spinal cord tissue has shown significant microglial and astrocytic activation in a female patient with longstanding complex regional pain syndrome [78]. In post-mortem tissue of male patients with HIV-associated neuropathic pain, astrocytic but not microglial markers are upregulated in the spinal dorsal horn [79]. In-vivo imaging of patients of both sexes with chronic low back pain shows evidence of glial activation in the brain [80]. Female and male patients with peripheral nerve injury display activation of glial cells in the thalamus [81]. Thus, there is evidence that glial cells, including microglia, are activated in certain chronic pain conditions. This glial reactivity is observed in patients of both sexes [78–81], which is consistent with the preclinical data [8]. Given the dissociation between microglial proliferation and pain hypersensitivity, it cannot be concluded that microglia in humans of either sex are involved chronic pain per se [40].

The preclinical literature on microglia and pain has sparked significant interest in targeting microglia in order to treat chronic pain in humans. However, a clinical trial of propentofylline, a glial modulating drug, failed to show efficacy in treating pain in patients with post-herpetic neuralgia [82]. It is possible that the dosing schedule of propentofylline may not have been sufficient to achieve adequate spinal glial inhibition. Additionally, post-herpetic neuralgia is not strictly a nerve lesion, which has been the primary preclinical model used to establish microglial involvement in pain processing. Alternatively, the clinical trial may indicate a lack of microglial involvement in neuropathic pain in humans. Whether the efficacy of propentofylline in treating neuropathic pain differs between women and men was not reported. Thus, a possible sexually dimorphic role of microglia in pain in humans cannot be ruled out.

## Conclusions

Microglia have become a focus in the preclinical pain research field. There is clear evidence in rodent models of neuropathic pain that microglia are critical in mediating pain behavior associated with nerve injury. However, the evidence implicating microglia in pain comes almost exclusively from experiments using male rodents. Recent research has put into question our understanding of the role of microglia in pain processing. While microglia adopt a reactive phenotype following nerve injury in mice of both sexes, these cells are not involved in mediating neuropathic pain behavior in females. The cross-species generalizability of this sexual dimorphism remains to be fully explored, but preliminary evidence supports the existence of sex differences in pain signalling in rats with nerve injury. Sexually dimorphic pain processing denotes the importance of including animals of both sexes in preclinical research. The translational potential of preclinical pain research may be greatly improved with equal representation of male and female subjects and may lead to development of precision medicine for chronic pain patients.

## Abbreviations

BDNF: Brain derived neurotrophic factor
CA: Carbonic anhydrase
CNS: Central nervous system
CSF1: Colony-stimulating factor 1
GABA_A_: γ-aminobutyric acid type A
KCC2: Potassium chloride cotransporter 2
ND2: NADH dehydrogenase subunit 2
MBP: Myelin basic protein
P2X4R: P2X4 receptor
PNI: Peripheral nerve injury
PPAR: Peroxisome proliferator activated receptor
